# Chemical Constituents from the Stems of *Tinospora sinensis* and Their Bioactivity

**DOI:** 10.3390/molecules23102541

**Published:** 2018-10-05

**Authors:** Sio-Hong Lam, Po-Hsun Chen, Hsin-Yi Hung, Tsong-Long Hwang, Chih-Chao Chiang, Tran Dinh Thang, Ping-Chung Kuo, Tian-Shung Wu

**Affiliations:** 1 School of Pharmacy, College of Medicine, National Cheng Kung University, Tainan 701, Taiwan; shlam@mail.ncku.edu.tw (S.-H.L.); z10308005@email.ncku.edu.tw (H.-Y.H.); 2 Department of Biotechnology, National Formosa University, Yunlin 632, Taiwan; pcckuoo@gmail.com; 3 Graduate Institute of Natural Products, School of Traditional Chinese Medicine, College of Medicine, Chang Gung University, Taoyuan 333, Taiwan; htl@mail.cgu.edu.tw; 4 Research Center for Chinese Herbal Medicine, Research Center for Food and Cosmetic Safety, Graduate Institute of Health Industry Technology, College of Human Ecology, Chang Gung University of Science and Technology, Taoyuan 333, Taiwan; 5 Department of Anesthesiology, Chang Gung Memorial Hospital, Taoyuan 333, Taiwan; 6 Graduate Institute of Clinical Medical Sciences, College of Medicine, Chang Gung University, Taoyuan 338, Taiwan; moonlight0604@hotmail.com; 7 Supervisor Board, Taoyuan Chinese Medicine Association, Taoyuan 338, Taiwan; 8 Dazhu Fengze Chinese Medicine Clinic, Taoyuan 338, Taiwan; 9 School of Chemistry, Biology and Environment, Vinh University, Vinh City 44000, Vietnam; thangtd@vinhuni.edu.vn; 10 NTT Institute of High Technology, Nguyen Tat Thanh University, Ho Chi Minh City 700000, Vietnam; 11 Department of Pharmacy, College of Pharmacy and Health Care, Tajen University, Pingtung 907, Taiwan

**Keywords:** Menispermaceae, lignan, pyrrole alkaloid, superoxide anion generation, elastase release

## Abstract

Fifty-seven compounds were purified from the stems of *Tinospora sinensis*, including three new compounds characterized as a lignan (**1**), a pyrrole alkaloid (**11**), and a benzenoid (**17**), respectively. Their structures were elucidated and established by various spectroscopic and spectrometric analytical methods. Among the isolates, fifteen compounds were examined for their anti-inflammatory potential in vitro. The results showed that several compounds displayed moderate inhibition of *N*-formyl-methionyl-leucyl-phenylalanine/cytochalasin B (fMLP/CB)-induced superoxide anion generation and elastase release.

## 1. Introduction

Inflammation is the first response of the immune system to infection or irritation. Neutrophils play an important role in eliminating most of the exogenous pathogens. Various autoimmune diseases are linked to neutrophil overexpression, such as rheumatoid arthritis, ischemia, and asthma, etc. [1,2,3]. According to response of diverse stimuli, activated neutrophils will secrete a series of cytotoxins. The superoxide anions and neutrophil elastase are the major secreted products of stimulated neutrophils in infected tissues and organs, which contribute to the destruction of tissue in chronic inflammatory diseases [4,5,6]. Therefore, inhibition of superoxide anion generation and elastase release by natural compounds is considered to be an effective screening platform to evaluate anti-inflammatory drug candidates.

**Figure 1:**
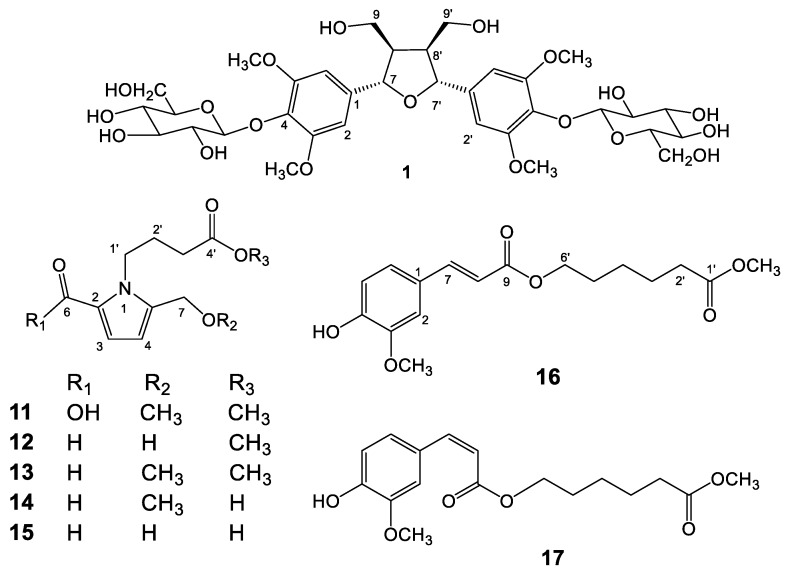
Structures of compounds **1** and **11**–**17**.

The genus *Tinospora*, belonging to family Menispermaceae, is composed of more than 20 species all over the tropical regions of the Eastern Hemisphere [7]. This genus is traditionally medical used in Southeast Asian countries for treating malaria, skin diseases, gout, and diabetes [8]. The majority of scientific reports of this genus state their physiological activities including antioxidation, anti-inflammation, and cytotoxicity, especially with the most extensively explored hypoglycemic activity [9,10,11,12,13]. However, the bioactive principles of *T. sinensis* remained poorly understood. Therefore, this plant was selected for study to discover novel anti-inflammatory lead compounds due to their relieving rigidity of muscles and activating collaterals effects in long-term folk medicine usage, which may be related to anti-inflammatory bioactivity. According to the preliminary screening results, the methanol extract of *T. sinensis* collected from Vietnam displayed half maximal inhibitory concentration (IC_50_) values of 6.66 μg/mL and 4.68 μg/mL in the inhibition of superoxide anion generation and elastase release, respectively (Table S1). Further chromatography purification resulted in the characterization of nine lignans (**1**–**9**), six pyrrole alkaloids (**10**–**15**), seventeen benzenoids (**16**–**32**), ten terpenoids (**33**–**42**), eight steroids (**43**–**50**), four amides (**51**–**54**), one coumarin (**55**), and two others (**56**–**57**), respectively. The chemical structures of new compounds **1**, **11**, and **17** (Figure 1) were established on the basis of nuclear magnetic resonance (NMR) and mass spectrometric analyses. Some of these purified compounds were examined for inhibition of superoxide anion generation and elastase release, thereby evaluating their in vitro anti-inflammatory potentials.

## 2. Results and Discussion

The dried stems of *T. sinensis* were refluxed with methanol and the obtained extract was divided into chloroform (CHCl_3_) and water (H_2_O) soluble fractions by liquid–liquid partition. Further purification over silica gel column and preparative thin layer chromatography (pTLC) resulted in the isolation of fifty-seven compounds. Among the isolated compounds, **1**, **11**, and **17** were new compounds. The other fifty-four known compounds were identified, including eight lignans, (+)-pinoresinol (**2**) [14], syringaresinol (**3**) [15], medioresinol (**4**) [16], (+)-*epi*-syringaresinol (**5**) [15], (+)-pinoresinol monomethyl ether (**6**) [17], (+)-glaberide I (**7**) [18], sesamin (**8**) [19], and sesamolin (**9**) [20]; five pyrrole alkaloids, 5-(hydroxymethyl)-1*H*-pyrrole-2-carbaldehyde (**10**) [21], methyl 4-[formyl-5-(hydroxymethyl)-1*H*-pyrrol-1-yl] butanoate (**12**) [22,23], methyl 4-[formyl-5-(methoxymethyl)-1*H*-pyrrol-1-yl] butanoate (**13**) [22,23], 4-[formyl-5-(methoxymethyl)-1*H*-pyrrol-1-yl] butanoic acid (**14**) [22,23], and 4-[formyl-5-(hydroxymethyl)-1*H*-pyrrol-1-yl] butanoic acid (**15**) [23]; seventeen benzenoids, rhodiolate (**16**) [24], methyl ferulate (**18**) [25], β-hydroxypropiovanillone (**19**) [26], 2-methyl-4,5-dimethoxybenzoic acid (**20**) [27], vanillic acid (**21**) [28], *p*-hydroxyl phenethanol (**22**) [29], tachioside (**23**) [30], icariside D_2_ (**24**) [31], salidroside (**25**) [32], syringin (**26**) [33], cordifolioside A (**27**) [34], *p*-hydroxybenzoic acid (**28**) [35], 4-(2-hydroxyethyl)benzoic acid (**29**) [36], syringic acid-4-*O*-α-l-rhamnoside (**30**) [37], isovanillic acid (**31**) [38], syringic acid (**32**) [39]; ten terpenoids, loliolide (**33**) [40], abscisic acid (**34**) [41], 3(17)-phytene 1,2-diol (**35**) [42], malabarolide (**36**) [43], lupeol (**37**) [44], 3-*O*-acetyloleanolic acid (**38**) [45], cycloeucalenol (**39**) [46], cycloabyssinone (**40**) [47], cycloartane-3β,25-diol (**41**) [48], and cycloart-22-ene-3β,25-diol (**42**) [49]; eight steroids, β-sitosterol (**43**) [50], stigmasterol (**44**) [50], 7α-hydroxysitosterol (**45**) [51], 7α-hydroxystigmasterol (**46**) [51], 6β-hydroxystigmast-4-en-3-one (**47**) [52], 6β-hydroxystigmasta-4,22-dien-3-one (**48**) [52], 7-ketositosterol (**49**) [53], and 3β-hydroxy-stigmasta-5,22-dien-7-one (**50**) [53]; four amides, 5,6-dimethoxy-*N*-methylphthalimide (**51**) [54], *N*-*trans*-feruloyldopamine (**52**) [55], *N*-*trans*-feruloyltyramine (**53**) [56], *N*-*cis*-feruloyltyramine (**54**) [57]; and one coumarin, scopoletin (**55**) [58]; and two others, lichexanthone (**56**) [59] and 2,6-dimethoxy-*p*-quinone (**57**) [60], respectively. The chemical structures of these new constituents were determined on the basis of 1D and 2D NMR and mass spectrometric analyses elucidated as follow.

**Figure 2:**
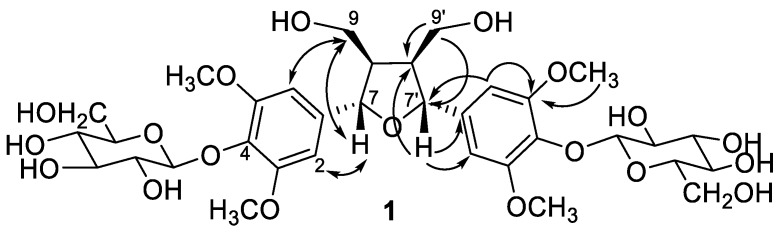
Diagnostic HMBC (→) and NOESY (↔) correlations of compound **1**.

The molecular formula of compound **1** was determined as C_34_H_48_O_19_ by high resolution electrospray ionization mass spectrometry (HR-ESI-MS) which showed a quasi-molecular ion peak [M − H − H_2_O]^−^ at *m*/*z* 741.2612. The ^1^H and ^13^C-NMR spectra (Table 1) revealed the presence of two sets of 1,3,4,5-tetrasubstituted symmetrical aromatic rings [δ_H_ 6.66 (H-2, 6, 2′, 6′) and δ_C_ 133.7 (C-1, 1′), 104.2 (C-2, 6, 2′, 6′), 152.6 (C-3, 5, 3′, 5′), 137.1 (C-4, 4′)], two oxymethylenes [δ_H_ 4.18 (dd, *J* = 9.0, 6.7 Hz), 3.84 (dd, *J* = 9.0, 3.2 Hz) and δ_C_ 71.3 (C-9, 9′)], two methines [δ_H_ 3.09 (m, H-8, 8′) and δ_C_ 53.6 (C-8, 8′)], two oxymethines [δ_H_ 4.66 (brd, *J* = 3.8, H-7, 7′) and δ_C_ 85.0 (C-9, 9′)], and two methoxy groups (δ_H_ 3.76 and δ_C_ 56.4). The correlation spectroscopy (COSY) spectrum provided key correlations between H-7 (δ_H_ 4.66) and H-8 (δ_H_ 3.09), and between H-8 (δ_H_ 3.09) and methylene H-9 protons (δ_H_ 4.18 and 3.84). Its heteronuclear multiple bond correlation (HMBC) spectrum provided further correlations from H-7 to C-1, C-2, C-6, and C-8 suggested the aromatic ring was attached to C-7 (Figure 2). From these spectral information, **1** was indicated as a 2,5-diaryl tetrahydrofuranoid type lignan. Two sets of β-glucopyranosyl unit [δ_H_ 4.90 (br d, *J* = 5.2 Hz) and δ_C_ 102.6 (G-1, 1′), δ_H_ 3.59, 3.40 and δ_C_ 102.6 (G-6, 6′)] were also observed. The glucosylation shifts at C-9, -9′ (δ_C_ 71.3) and C-8, -8′ (δ_C_ 53.6) constructed the location of the glucosyl units at C-9 and C-9′ of the aglycone, when compared with unbound C-9 (δ_C_ 61.2) and C-8 (δ_C_ 54.9) reported in the literature [61]. The relative configurations between C-7 and C-8 (also C-7′ and C-8′) were established as *trans*-configurations due to no nuclear Overhauser effect (NOE) correlations between H-7 and H-8 (also H-7′ and H-8′) in the nuclear Overhauser enhancement spectroscopy (NOESY) experiment (Figure 2). Thus, the structure of compound **1** was determined as dihydroxymethylbis(3,5-dimethoxy-4-hydroxyphenyl)tetrahydrofuran-9,9′-*O*-β-diglucopyranoside and named trivially as tinosporide A.

**Figure 3:**
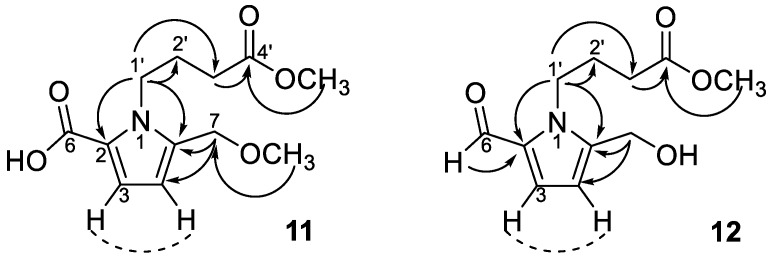
Diagnostic HMBC (→)/COSY (---) correlations of compounds **11** and **12**.

Compounds **11**–**15** all exhibited similar ultraviolet (UV) and infrared (IR) absorption characteristics. Their UV spectra all displayed absorption maxima close to 293 nm, which are characteristic of the pyrrole-2-carbonyl basic skeleton [62]. The ^1^H-NMR spectrum (Table 2) exhibited signals for two methine protons at δ_H_ 6.16 (d, *J* = 3.9 Hz, H-4) and 7.01 (d, *J* = 3.9 Hz, H-3). Chemical shifts at δ_C_ 110.8 (C-4), 119.0 (C-3), 121.6 (C-2), and 136.9 (C-5) in ^13^C-NMR spectrum implied the occurrence of a heterocyclic ring containing a nitrogen atom and their proton coupling constants also indicated the 2,5 di-substituted pyrrole ring (Table 2). The ^1^H and ^13^C-NMR spectra of **11** also evidenced the presence of a butanoic acid moiety which appeared at δ_H_ 4.37 (br t, *J* = 7.6 Hz, H-1′), 2.36 (t, *J* = 7.3 Hz, H-3′), and 2.04 (m, H-2′), confirmed by HMBC correlations from H-3′ and H-2′ to a carbonyl carbon (δ_C_ 173.4, C-4′). The connection of the butanoic acid moiety on the nitrogen atom was suggested by observing long range correlation peaks from δ_H_ 4.37 (H-1′) to δ_C_ 136.9 (C-5) and δ_C_ 121.6 (C-2) in the HMBC spectrum (Figure 3). These spectral data clearly determined that a butanoic acid moiety was attached to N-1 of the pyrrole ring. An oxomethylene group connected to C-5 of pyrrole ring was proved by the HMBC correlation of δ_H_ 4.43 (H-7) and δ_C_ 136.9 (C-5). Two additional methoxy groups (δ_H_ 3.34, δ_C_ 51.6; δ_H_ 3.67, δ_C_ 57.7) were also observed and deduced to be located at C-7 and C-4′ by HMBC analysis (Figure 3). However, the HR-ESI-MS analytical data was unavailable due to the sample lability. Therefore, the molecular formula of **11** was proposed as C_12_H_17_NO_5_ according to the above-mentioned NMR spectral analysis and gas chromatograph–mass spectrometer (GC–MS) analytical results which exhibited a molecular ion peak at *m*/*z* 255 (see Supplementary Materials). On the basis of these data, the structure of **11** was determined as 1-(4-methoxy-4-oxobutyl)-5-(methoxymethyl)-1*H*-pyrrole-2-carboxylic acid and named trivially as tinosporin A. 

Compound **12** displayed very similar ^1^H and ^13^C-NMR signals (Table 2) as those of **11** except an additional aldehyde signal (δ_H_ 9.42 (*s*, H-6) and δ_C_ 180.9 (C-6)) and one methoxy group (δ_H_ 3.66 (*s*, OCH_3_) and δ_C_ 52.2 (OCH_3_)). Its HMBC spectrum exhibited the correlations from methoxy protons to butanoic acid C-4′ (δ_C_ 175.1), as shown in Figure 3. The molecular formula of **12** was proposed as C_11_H_15_NO_4_ also based on the GC–MS analytical data of the molecular ion peak at *m*/*z* 225 (see Supplementary Materials). Accordingly, the structure of **12** was established as methyl 4-[formyl-5-(hydroxymethyl)-1*H*-pyrrol-1-yl] butanoate. Compound **13** was shown to possess the molecular formula of C_12_H_17_O_4_N by GC–MS measurement. An additional methoxy group (δ_H_ 3.36) was observed in **13** by comparison of its ^1^H-NMR spectra with that of **12**. The structure of compound **13** was elucidated as a methyl 4-[formyl-5-(methoxymethyl)-1*H*-pyrrol-1-yl] butanoate. Furthermore, compounds **14** and **15** were determined as 4-[formyl-5-(methoxymethyl)-1*H*-pyrrol-1-yl] butanoic acid and 4-[formyl-5-(hydroxymethyl)-1*H*-pyrrol-1-yl] butanoic acid, respectively, by comparison of their spectral data with those reported [22,23]. According to the above results, pyrrole alkaloids **10–15** were reported from *Tinospora* genus for the first time.

**Figure 4:**
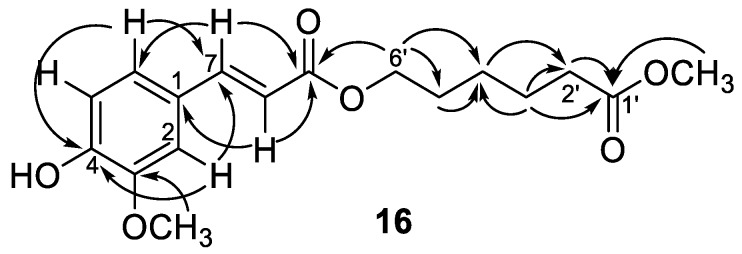
Diagnostic HMBC (→) correlations of compound **16**.

Compounds **16** and **17** showed the same adduct ion peaks and were both assigned the same molecular formula C_17_H_22_O_6_. The ^1^H-NMR spectrum of **16** revealed the existence of an aromatic protons at δ_H_ 7.07 (dd, *J* = 8.2, 1.8 Hz, H-6), 7.04 (d, *J* = 1.8 Hz, H-2), and 6.92 (d, *J* = 8.2 Hz, H-5); five methylenes at δ_H_ 4.19 (t, *J* = 6.6 Hz, H-6′), 2.34 (t, *J* = 7.4 Hz, H-2′), 1.67 (m, H-3′, 5′), and 1.47 (m, H-4′); and two methoxy singlets at δ_H_ 3.95 and 3.67. Additional signals at δ_H_ 7.59 (d, *J* = 16.0 Hz, H-7) and 6.47 (d, *J* = 16.0 Hz, H-8) suggested the presence of a *trans* double bond. The ^13^C-NMR spectrum revealed the existence of seventeen carbon atoms included an aromatic ring (δ_C_ 109.3, 112.7, 123.1, 127.0, 146.6, and 147.9), five methylenes (δ_C_ 64.0, 33.8, 28.1, 25.3, and 24.5), two methoxyls (δ_C_ 55.9 and 51.5), two carbonyls (δ_C_ 173.9 and 167.5), and a pair of olefinic carbons (δ_C_ 144.8 and 115.5). A 3,4-disubstituted cinnamoyl group linked with a hexanoyl alcohol was deduced from the NMR data which described above (Table 1). This was further confirmed by the key HMBC correlations from δ_H_ 3.67 (OCH_3_) to 173.9 (C-1′), from δ_H_ 4.19 (H-6′) to δ_C_ 167.5 (C-9), and 28.1 (C-5′), as shown in Figure 4. Therefore, compound **16** was confirmed as rhodiolate by comparison of its spectral data with those reported [24]. Compound **17** displayed closely related 1D NMR spectroscopic and mass spectrometric characteristics to **16** and was determined to have a similar structure to **16**. However, a pair of olefinic protons at δ_H_ 6.80 (d, *J* = 12.9 Hz, H-7) and 5.81 (d, *J* = 12.9 Hz, H-8) suggested the *cis* double bond feature. However, 2D NMR spectral analysis of **17** could not be furnished because of the rapid transformation of *cis–trans* double bond. Thus, the structure of compound **17** was concluded to be methyl 6-((Z)-3-(4-hydroxy-3-methoxyphenyl)acryloyloxy)-hexanoate and assigned the trivial name as tinosporin B.

Fifteen purified compounds were examined for their inhibition bioactivity of superoxide anion generation and elastase release by human neutrophils in response to fMLP/CB (Table S2) [63,64]. However, most displayed weak inhibition percentages at the test concentration (10 μM). Among these, **1**, **16**, and **17** displayed higher inhibitions of superoxide anion generation at 10 μM with inhibition percentages ranged from 10.2 ± 7.1 to 20.2 ± 5.1%. In addition, compound **39** (10 μM) also exhibited inhibitory effect on elastase release with inhibition percentage of 22.3 ± 10.0% (Table S2). Columbin, an important furanoditerpenoid isolated from several *Tinosporae Radix*, exhibited significant anti-inflammatory activities in a dose-dependent manner [65]. However, based on our research data the related furanoid bisnorditerpenoid, malabarolide (**36**), was not the predominant component, maybe due to the different parts of plant materials. The conventional use of *T. sinensis* in traditional Chinese medicine is for relieving rigidity of muscles and activating collaterals, and the mechanism of action may be related to anti-inflammatory bioactivity. The present experimental data not only suggest that the extracts and purified compounds of the stems of *T. sinensis* have the potential to be developed as novel anti-inflammatory lead drugs or health foods, but also merit further investigation of the anti-inflammatory mechanism.

## 3. Materials and Methods 

### 3.1. General Information

Optical rotations and UV spectra were measured using a Atago AP-300 digital polarimeter (Atago, Tokyo, Japan) and a GBC Cintra 101 spectrophotometer (GBC Scientific Equipment Ltd., Dandenong, Australia), respectively. IR spectra were obtained with a Shimadzu FT-IR Prestige-21 spectrophotometer (Shimadzu, Kyoto, Japan). ^1^H and ^13^C-NMR spectra were recorded on Bruker AV 700, AV 500, and Avance III 400 NMR spectrometers (Bruker, Billerica, MA, USA). Chemical shifts are shown in δ values (ppm) with tetramethylsilane as an internal standard. GC–MS were analyzed using a Shimadzu GC-2010 gas chromatograph/mass spectrometer equipped with a quadrupole mass analyzer (Shimadzu, Kyoto, Japan). The HR-ESI-MS were taken on a Bruker Daltonics micrOTOF orthogonal ESI-TOF mass spectrometer (Bruker, Billerica, MA, USA). Column chromatography (CC) was performed on silica (70–230 mesh and 230–400 mesh, Merck, Darmstadt, Germany) and Diaion HP-20 (Mitsubishi, Tokyo, Japan) gels, and preparative thin-layer chromatography (TLC) was conducted on Merck precoated silica gel 60 F254 plates (Merck, Darmstadt, Germany), using UV light to visualize the spots. Methanol, chloroform (GR grade), *n*-hexane, ethyl acetate, benzene, and acetone (ACS grade) were purchased from Merck (Darmstadt, Germany) and Mallinckrodt (St. Louis, MO, USA), respectively. DMSO-*d*_6_, CD_3_OD, and CDCl_3_ were purchased from Sigma-Aldrich (St. Louis, MO, USA).

### 3.2. Materials

The stems of *T. sinensis* were collected from Vietnam in August 2009, and the plant material was identified and authenticated by Assoc. Prof. Dr. Vu Xuan Phuong, Institute of Ecology and Biological Resources, Vietnamese Academy of Science and Technology. A voucher specimen (Viet-TSWu-2009-1801-001) was deposited in the herbarium of the Institute of Ecology and Biological Resources, Vietnamese Academy of Science and Technology, Hanoi, Vietnam. 

### 3.3. Extraction and Isolation

The dried stems of *T. sinensis* (10 kg) was refluxed with methanol (30 L × 8 × 8 h) and then filtered and concentrated under reduced pressure to obtain the methanol extract (400 g). The extract was suspended in distilled water and successively partitioned with chloroform to yield a chloroform layer (60 g) and water soluble (340 g). The chloroform layer was chromatographed directly on silica gel and eluted with a gradient of *n*-hexane and acetone to afford 10 fractions (CF 1-10). Fractions CF 1, 2, and 4 did not show any significant spots under TLC check and therefore were not purified further. Fraction CF 3 was isolated by CC on silica gel with a step gradient with benzene and acetone mixtures and the subfraction CF 3-6 was further purified by TLC using *n*-hexane-ethyl acetate (50:1) to yield cycloabyssinone (**40**, 3 mg). Fraction CF 5 was purified using silica gel CC eluted with gradient mixtures of *n*-hexane and acetone to afford thirteen subfractions (CF 5-1 to 5-13). CF 5-2 was fractionated by silica gel CC eluted with benzene ethyl acetate and then lupeol (**37**, 8 mg), cycloeucalenol (**39**, 15 mg), and a mixture of β-sitosterol (**43**) and stigmasterol (**44**) (364 mg), respectively, was purified from the minor fractions by TLC using *n*-hexane-ethyl acetate (50:1). CF 5-5 was performed on silica gel CC with gradient mixtures of hexane and acetone to produce ten minor fractions. One minor fraction CF 5-5-7 was purified by silica gel CC with mixture of benzene and acetone and further purification by TLC using chlorofrom-acetone (9:1) yielded a mixture of 7α-hydroxysitosterol (**45**) and 7α-hydroxystigmasterol (**46**) (6 mg). CF 5-7 was subjected to silica gel CC eluted with a gradient mixture of benzene ethyl acetate to afford ten minor fractions. CF 5-7-4 was further isolated by silica gel CC, eluted with hexane ethyl acetate and subsequent TLC using hexane ethyl acetate (6:1) to afford 3-*O*-acetyloleanolic acid (**38**, 4 mg).

Fraction CF 6 was isolated by silica gel CC by gradient elution with mixture of *n*-hexane and ethyl acetate to result in eleven subfractions (CF 6-1 to 6-11). CF 6-4 was further purified by silica gel CC eluted with *n*-hexane-acetone to produce eight minor fractions (CF 6-4-1 to 6-4-8). Lichexanthone (**56**, 4 mg) was purified by TLC using chloroform-ethyl acetate (100:1) from CF 6-4-3. CF 6-4-4 was subjected to silica gel CC eluted by benzene-acetone gradient mixtures and further purified by TLC using chloroform:acetone (10:1) to afford 2-methyl-4,5-dimethoxybenzoic acid (**20**, 4 mg). CF 6-5 was subjected to silica gel CC with chloroform and methanol gradient mixtures to afford five minor fractions. CF 6-5-2 was isolated by silica gel CC eluted by chloroform:ethyl acetate gradient mixtures and subsequent TLC using hexane-ethyl acetate (10:1) to produce tinosporin A (**11**, 1 mg), 3(17)-phytene 1,2-diol (**35**, 3 mg), cycloart-22-ene-3β,25-diol (**42**, 4 mg), 5,6-dimethoxy- *N*-methyl-phthalimide (**51**, 8 mg), respectively. CF 6-6 was isolated by silica gel CC with chloroform and methanol gradient mixtures and further purified by TLC using hexane:acetone (10:1) to yield methyl 4-[formyl-5-(methoxymethyl)-1*H*-pyrrol-1-yl] butanoate (**13**, 2 mg).

Fraction CF 7 was chromatographed on silica gel column eluted with gradient mixtures of chloroform and ethyl acetate to afford seven subfractions (CF 7-1 to 7-7). CF 7-2 was purified by silica gel CC successively eluted with hexane:acetone, hexane ethyl acetate, and chloroform ethyl acetate and one minor fraction (CF 7-2-5-3) to afford methyl ferulate (**18**, 5 mg). Another minor fraction CF 7-2-5-4 was further isolated by silica gel CC with gradient elution of benzene and acetone, and subsequent purification by TLC using hexane ethyl acetate (5:1) to give rhodiolate (**16**, 2 mg) and tinosporin B (**17**, 2 mg). CF 7-3 was also performed silica gel CC eluted with hexane ethyl acetate to afford ten minor fractions, and CF 7-3-7 was further isolated by silica gel CC eluted with hexane-ethyl acetate and subsequent TLC using benzene ethyl acetate (30:1) to afford (+)-pinoresinol monomethyl ether (**6**, 3 mg). CF 7-4 was isolated by silica gel CC eluted with hexane ethyl acetate to yield ten minor fractions. Of these, CF 7-4-5 was further purified by silica gel CC (hexane-acetone mixing eluents) and subsequent TLC using chloroform:acetone (20:1) to afford cycloartane-3β,25-diol (**41**, 16 mg). CF 7-4-6 was also subjected into silica gel CC (hexane:acetone mixing eluents) to give seven minor fractions. Further purification of CF 7-4-6-4, CF 7-4-6-5, and CF 7-4-6-6 by silica gel CC eluted with chloroform:acetone (9:1) to yield loliolide (**33**, 5 mg), a mixture of 6β-hydroxystigmast-4-en-3-one (**47**) and 6β-hydroxystigmasta-4,22-dien-3-one (**48**) (2 mg), and a mixture of 7-ketositosterol (**49**) and 3β-hydroxystigmasta-5,22-dien-7-one (**50**) (6 mg), respectively.

Fraction CF 8 was isolated by silica gel CC eluted with gradient mixtures of hexane and acetone to afford six subfractions (CF 8-1 to 8-6). CF 8-4 was performed silica gel CC eluted with hexane ethyl acetate and further purified by TLC using benzene:acetone (20:1) to give *N*-*trans*-feruloyldopamine (**52**, 6 mg). Ten subfractions (CF 9-1 to 9-10) were obtained from CF 9 by silica gel CC eluted with gradient mixture of chloroform and acetone. CF 9-3 was further isolated by silica gel CC, eluted with benzene:ethyl acetate and, following TLC purification of minor fraction CF 9-3-6 using chloroform:acetone (30:1) to afford (+)-pinoresinol (**2**, 10 mg) and scopoletin (**55**, 3 mg), CF 9-3-7 was further purified by TLC using chloroform:acetone (10:1) to afford medioresinol (**4**, 4 mg), (+)-epi-syringaresinol (**5**, 3 mg), (+)-glaberide I (**7**, 3 mg), and 2,6-dimethoxy-p-quinone (**57**, 5 mg), respectively. CF 9-3-8 was isolated by silica gel CC eluted with gradient mixtures of chloroform-methanol and then purified by TLC using chloroform:methanol (300:1) to yield syringaresinol (**3**, 12 mg). CF 9-4 was divided to eight minor fractions by silica gel CC eluted with benzene:acetone solvent mixture. Of these, CF 9-4-5 was further fractionated by silica gel CC eluted with chloroform:acetone (30:1) to give β-hydroxypropiovanillone (**19**, 3 mg). CF 9-7 was isolated by silica gel CC (chloroform:acetone gradient mixture) to yield six minor fractions and one of these CF 9-7-4 was afforded *N*-*trans*-feruloyltyramine (**53**, 8 mg) and *N*-*cis*-feruloyltyramine (**54**, 5 mg) by further silica gel CC eluted with chloroform:acetone (30:1) and subsequent TLC using chloroform:methanol (50:1). The last fraction (CF 10) of the chloroform layer was also purified by silica gel CC eluted with gradient mixture of chloroform and acetone. The resulting subfraction CF 10-5 was divided to several minor fractions by silica gel CC eluted with chloroform:methanol (50:1) solvent mixture and further purified by TLC using chloroform:acetone (10:1) to give abscisic acid (**34**, 1 mg).

The water soluble fraction was subjected directly to Diaion HP-20 column chromatography, eluted by water and gradient with methanol, to afford seventeen fractions (WF 1-17). Fractions WF 1-5, 9, 11, and 14-16 did not show any significant spots under TLC check and therefore were not purified further. WF 6, 7, and 8 were purified by silica gel CC eluted with gradient mixture of chloroform and methanol and afforded tachioside (**23**, 10 mg); vanillic acid (**21**, 5 mg), *p*-hydroxyl phenethanol (**22**, 3 mg), icariside D_2_ (**24**, 10 mg); and salidroside (**25**, 10 mg), respectively.

Fraction WF 10 was chromatographed on silica gel column eluted with gradient mixtures of chloroform and methanol to afford six subfractions (WF 10-1 to 10-6). WF 10-2 was purified by silica gel CC eluted with chloroform and methanol and one minor fraction (WF 10-2-3) affording 4-(2-hydroxyethyl)benzoic acid (**29**, 2 mg). WF 10-3 was also performed silica gel CC eluted with chloroform and methanol solvent mixture to afford ten minor fractions, and WF 10-3-4 was further isolated by silica gel CC eluted with chloroform and acetone (10:1) to afford *p*-hydroxybenzoic acid (**28**, 5 mg). WF 10-4 was isolated by silica gel CC eluted with chloroform and methanol solvent mixture to yield ten minor fractions. Of these, WF 10-4-5 was further purified by silica gel CC (chloroform:acetone mixing eluents) and subsequent TLC using chloroform:acetone (10:1) to afford 5-(hydroxymethyl)-1*H*-pyrrole-2-carbaldehyde (**10**, 1 mg). Recrystallization of WF 10-4-7 and 10-4-9 by chloroform:acetone produced syringin (**26**, 25 mg) and cordifolioside A (**27**, 30 mg), respectively. WF 10-6 was isolated by silica gel CC eluted with chloroform and methanol solvent mixture to yield five minor fractions. Of these, WF 10-6-3 was further purified by silica gel CC eluted by chloroform and acetone (9:1) to afford syringic acid-4-*O*-α-l-rhamnoside (**30**, 8 mg).

Fractions WF 12, 13, and 17 were all chromatographed on silica gel column eluted with gradient mixtures of chloroform and methanol to produce several subfractions. WF 12-2 was purified by silica gel CC eluted with chloroform ethyl acetate to afford methyl 4-[formyl-5-(hydroxymethyl)-1*H*-pyrrol-1-yl] butanoate (**12**, 10 mg). Similarly, 4-[formyl-5-(methoxymethyl)-1*H*-pyrrol-1-yl] butanoic acid (**15**, 5 mg) and 4-[formyl-5-(methoxymethyl)-1*H*-pyrrol-1-yl] butanoic acid (**14**, 7 mg) resulted from the chromatographic elution of WF 12-5 and 12-12, respectively. WF 13-1 was isolated by silica gel CC eluted with chloroform and methanol solvent mixture to yield ten minor fractions. Of these, WF 13-1-7 was further purified by silica gel CC (chloroform-acetone mixing eluents) and subsequent TLC using chloroform:acetone (10:1) to syringic acid (**32**, 3 mg). Another subfraction WF 13-3 was further isolated by silica gel CC with gradient elution of chloroform and methanol, and subsequent purification by TLC using chloroform and methanol (9:1) to give isovanillic acid (**31**, 2 mg). Recrystallization of WF 13-4 and 13-13 by chloroform:acetone produced tinosporide A (**1**, 15 mg) and malabarolide (**36**, 10 mg), respectively. WF 17-2 was isolated by silica gel CC eluted with chloroform and methanol (9:1) and further purified by TLC using chloroform:acetone (20:1) to afford sesamin (**8**, 5 mg) and sesamolin (**9**, 2 mg).

*Tinosporide A* (**1**): colorless powder; UV (MeOH) λ _max_ (log ε) 272 (2.87) nm; IR (neat) ν_max_ 3258, 2862, 2358, 1592, 1457, 1418, 1235, 1131, 1045 cm^−1^; ^1^H-NMR (500 MHz, DMSO-*d*_6_) and ^13^C-NMR (125 MHz, DMSO-*d*_6_), see Table 1; HR-ESI-MS *m/z* 741.2612 ([M − H − H_2_O]^−^, calcd for C_34_H_45_O_18_, 741.2611).

*Tinosporin A* (**11**): Pale yellow syrup; UV (EtOH) λ_max_: 319, 293, 220 nm; ^1^H-NMR (700 MHz, CDCl_3_) and ^13^C-NMR (175 MHz, CDCl_3_), see Table 2; GC–MS *m*/*z* 255 ([M]^+^), 237, 210, 180, 136, 101, 59.

*Tinosporin B* (**17**): Colorless syrup; UV (MeOH) λ_max_ (log *ε*): 323 (3.32), 299 (3.18, sh), 235(3.13), 218(3.20) nm; IR (neat) ν_max_: 3410, 2926, 2853, 1729, 1709, 1632, 1595, 1515, 1464, 1432, 1376, 1270, 1162, 1126, 1033 cm^−1^; ^1^H-NMR (400 MHz, CDCl_3_) see Table 1; HR-ESI-MS *m*/*z* 345.1311 ([M + Na]^+^, calcd for C_17_H_22_O_6_Na, 345.1309).

### 3.4. Anti-inflammatory Bioactivity Examination

#### 3.4.1. Preparation of Human Neutrophils

The use of human neutrophils was approved by the Institutional Review Board at Chang Gung Memorial Hospital, Taoyuan, Taiwan, and the study was conducted according to the Declaration of Helsinki (2013). Written informed consent was obtained from each healthy donor before blood was drawn. The details of the preparation of human neutrophils are provided in the Supplementary Materials.

#### 3.4.2. Measurement of Superoxide Anion Generation and Elastase Release

The assay of the generation of superoxide anion was based on the superoxide dismutase (SOD)-inhibitable reduction of ferricytochrome c. Degranulation of azurophilic granules was determined by elastase release as described previously [63,64]. The details of measurement of superoxide anion generation and elastase release were provided in the Supplementary Materials.

## Figures and Tables

**Table 1:** NMR Spectroscopic Data of Compounds **1**, **16**, and **17**.

Position	1 *^a^*			17 *^b^*	16 *^b^*	
	*δ*_H_	*δ*_C_	*HMBC (H*→*C)*	*δ*_H_	*δ*_H_	*δ*_C_
1		133.7 *s*				127.0 *s*
2	6.66 *s*	104.2 *d*	85.0, 104.2, 137.1, 152.6	7.76 *d* (1.9)	7.04 *d* (1.8)	109.3 *d*
3		152.6 *s*				146.6 *s*
4		137.1 *s*				147.9 *s*
5		152.6 *s*		6.88 *d* (8.3)	6.92 *d* (8.2)	112.7 *d*
6	6.66 *s*	104.2 *d*	85.0, 104.2, 137.1, 152.6	7.10 *dd* (8.3, 1.9)	7.07 *dd* (8.2, 1.8)	123.1 *d*
7	4.66 br *d* (3.8)	85.0 *d*	53.6, 71.3, 104.2, 137.1	6.80 *d* (12.9)	7.59 *d* (16.0)	144.8 *d*
8	3.09 *m*	53.6 *d*		5.81 *d* (12.9)	6.28 *d* (16.0)	115.5 *d*
9	3.84 *dd* (9.0, 3.2)	71.3 *t*	53.6, 85.0			167.5 *s*
	4.18 *dd* (9.0, 6.7)		53.6, 85.0, 104.2			
1′		133.7 *s*				173.9 *s*
2′	6.66 *s*	104.2 *d*	85.0, 104.2, 137.1, 152.6	2.31 *t* (7.6)	2.34 *t* (7.4)	33.8 *t*
3′		152.6 *s*		1.66 *m*	1.67 *m*	24.5 *t*
4′		137.1 *s*		1.37 *m*	1.47 *m*	25.3 *t*
5′		152.6 *s*		1.66 *m*	1.67 *m*	28.1 *t*
6′	6.66 *s*	104.2 *d*	85.0, 104.2, 137.1, 152.6	4.12 *t* (6.6)	4.19 *t* (6.6)	64.0 *t*
7′	4.66 br *d* (3.8)	85.0 *d*	53.6, 71.3, 104.2, 137.1			
8′	3.09 *m*	53.6 *d*				
9′	3.84 *dd* (9.0, 3.2)	71.3 *t*	53.6, 85.0			
	4.18 *dd* (9.0, 6.7)		53.6, 85.0, 104.2			
Bz-OMe	3.76 s	56.4 *q*		3.93 *s*	3.95 *s*	55.9 *q*
OMe				3.67 *s*	3.67 *s*	51.5 *q*
Glc H1, 1′	4.90 d (5.2)	102.6 *d*	76.5, 74.1			
Glc H2, 2′	3.17 m	76.5 *d*	74.1			
Glc H3, 3′	3.17 m	74.1 *d*	76.5			
Glc H4, 4′	3.11 m	69.9 *d*	76.5			
Glc H5, 5′	3.02 m	77.2 *d*	69.9			
Glc H6, 6′	3.40 m	60.9 *t*	77.2			
	3.59 m					

*^a^*^1^H and ^13^C-NMR data measured in deuterated dimethyl sulfoxide (DMSO-*d*_6_) at 500 MHz and 125 MHz, respectively; *^b^*^1^H and ^13^C-NMR data measured in deuterated chloroform (CDCl_3_) at 400 MHz and 100 MHz, respectively.

**Table 2:** NMR spectroscopic data of compounds **11**–**15**.

Position	11 ^a^		12 ^b^		13 ^a^	14 ^c^	15 ^c^
	*δ*_H_	*δ*_C_	*δ*_H_	*δ*_C_	*δ*_H_	*δ*_H_	*δ*_H_
2		121.6 *s*		133.5 *s*			
3	7.01 *d* (3.9)	119.0 *d*	6.98 *d* (4.0)	126.5 *d*	6.87 *d* (4.0)	6.96 *d* (4.0)	6.97 *d* (4.0)
4	6.16 *d* (3.9)	110.8 *d*	6.26 *d* (4.0)	111.5 *d*	6.23 *d* (4.0)	6.27 *d* (4.0)	6.25 *d* (4.0)
5		136.9 *s*		144.6 *s*			
6		162.2 *s*	9.42 *s*	180.9 *d*	9.50 *s*	9.45 *s*	9.40 *s*
7	4.43 *s*	65.8 *t*	4.63 *s*	56.4 *t*	4.45 *s*	4.52 *s*	4.65 *s*
1′	4.37 *br t* (7.6)	44.7 *t*	4.38 *dd* (7.4, 6.0)	45.7 *t*	4.36 *br t* (7.6)	4.35 *br t* (7.6)	4.37 *dd* (7.5, 6.0)
2′	2.04 m	26.5 *t*	2.01 m	27.5 *t*	2.01 m	1.96 m	1.98 m
3′	2.36 *t* (7.3)	31.0 *t*	2.35 *t* (7.3)	31.6 *t*	2.36 *t* (7.2)	2.23 *t* (7.5)	2.27 *t* (7.5)
4′		173.4 *s*		175.1 *s*			
OCH_3_	3.67 *s*	57.7 *q*	3.66 *s*	52.2 *q*	3.68 *s*		
CH_2_OCH_3_	3.34 *s*	51.6 *q*			3.36 *s*	3.36 *s*	

^1^H and ^13^C-NMR data measured in *^a^* CDCl_3_ at 400 MHz and 100 MHz; *^b^* CD_3_OD at 400 MHz and 100 MHz; *^c^* CD_3_OD at 500 MHz and 125 MHz, respectively.

## Supplementary Materials

The following are available online. S1: Anti-inflammatory bioactivity experimental procedures; Tables S1 and S2: Inhibitory effects of extracts and compounds from *T. sinensis*; Figures S1–S16: NMR spectra of compounds **1**, **11**, **12**, **16**, and **17**.
