# Supplementary material for: Chemical Constituents from the Stems of Tinospora sinensis and Their Bioactivity
Source: Molecules. 2018 Oct 5;23(10):2541. doi: 10.3390/molecules23102541 (PMC6222598; doi:10.3390/molecules23102541)
Supplement: Supplementary file 1 [file molecules-23-02541-s001.pdf]

Supporting Information

Chemical Constituents from the Stems of *Tinospora sinensis* and Their Bioactivity

Sio-Hong Lam [1], Po-Hsun Chen [2], Hsin-Yi Hung [1], Tsong-Long Hwang [3,4,5], Chih-Chao Chiang [6,7,8], Tran-Dinh Thang [9,10], Ping-Chung Kuo [1,*] and Tian-Shung Wu [1,11,*]

[1] School of Pharmacy, College of Medicine, National Cheng Kung University, Tainan 701, Taiwan; shlam@mail.ncku.edu.tw (S.-H.L.); z10308005@email.ncku.edu.tw (H.-Y.H.)

[2] Department of Biotechnology, National Formosa University, Yunlin 632, Taiwan; pcckuoo@gmail.com

[3] Graduate Institute of Natural Products, School of Traditional Chinese Medicine, College of Medicine, Chang Gung University, Taoyuan 333, Taiwan

[4] Research Center for Chinese Herbal Medicine, Research Center for Food and Cosmetic Safety, Graduate Institute of Health Industry Technology, College of Human Ecology, Chang Gung University of Science and Technology, Taoyuan 333, Taiwan

[5] Department of Anesthesiology, Chang Gung Memorial Hospital, Taoyuan 333, Taiwan; htl@mail.cgu.edu.tw

[6] Graduate Institute of Clinical Medical Sciences, College of Medicine, Chang Gung University; Taoyuan 338, Taiwan

[7] Supervisor board, Taoyuan Chinese Medicine Association; Taoyuan 338, Taiwan

[8] Dazhu Fengze Chinese Medicine Clinic, Taoyuan 338, Taiwan; moonlight0604@hotmail.com

[9] School of Chemistry, Biology and Environment, Vinh University, Vinh City 44000, Vietnam;

[10] NTT Institute of High Technology, Nguyen Tat Thanh University, Ho Chi Minh City 700000, Vietnam; thangtd@vinhuni.edu.vn

[11] Department of Pharmacy, College of Pharmacy and Health Care, Tajen University, Pingtung 907, Taiwan

* Correspondence: z10502016@email.ncku.edu.tw (P.-C.K.); tswu@mail.ncku.edu.tw (T.-S.W.); Tel.: +886-6-2353535 (ext. 6806) (P.-C.K.); Tel.: +886-6-2757575 (ext. 65333) (T.-S.W.)

Contents

S1. Anti-inflammatory Bioactivity Experimental Procedures

Table S1. Inhibitory Effects of extracts from *T. sinensis* on Superoxide Anion Generation and Elastase Release by Human Neutrophils in Response to fMLP/CB

Table S2. Inhibitory Effects of Isolated Compounds on Superoxide Anion Generation and Elastase Release by Human Neutrophils in Response to fMLP/CB

Fig. S1. ^{1}H NMR spectrum of **1**

Fig. S2. ^{13}C NMR spectrum of **1**

Fig. S3. HMBC spectrum of **1**

Fig. S4. NOESY spectrum of **1**

Fig. S5. ^{1}H NMR spectrum of **11**

Fig. S6. ^{13}C NMR spectrum of **11**

Fig. S7. COSY spectrum of **11**

Fig. S8. HMBC spectrum of **11**

Fig. S9. ^{1}H NMR spectrum of **12**

Fig. S10. ^{13}C NMR spectrum of **12**

Fig. S11. COSY spectrum of **12**

Fig. S12. HMBC spectrum of **12**

Fig. S13. ^{1}H NMR spectrum of **16**

Fig. S14. ^{13}C NMR spectrum of **16**

Fig. S15. HMBC spectrum of **16**

Fig. S16. ^{1}H NMR spectrum of **17**

Fig. S17. HR-MS spectrum of **1**

Fig. S18. HR-MS spectrum of **17**

Fig. S19. GC-MS spectrum of **11**

Fig. S20. GC-MS spectrum of **12**

S1. Anti-inflammatory Bioactivity Experimental Procedures

Preparation of Human Neutrophils. A study involving human neutrophils was approved by the Institutional Review Board at Chang Gung Memorial Hospital, Taoyuan, Taiwan, and was conducted according to the Declaration of Helsinki (2013). The written informed consent was obtained from each healthy donor before blood was drawn. Blood was drawn from healthy human donors (20–30 years old) by venipuncture into heparin-coated vacutainer tubes, using a protocol approved by the Institutional Review Board at Chang Gung Memorial Hospital. Blood samples were mixed gently with an equal volume of 3 % dextran solution. Neutrophils were isolated with a standard method of dextran sedimentation prior to centrifugation in a Ficoll Hypaque gradient and hypotonic lysis of erythrocytes. The leukocyte-rich plasma was collected after sedimentation of the red cells for 30 min at room temperature, and was transferred to 20 mL Ficoll solution (1.077 g/mL) and spun down at 400 g for 40 min at 20 °C. The granulocyte/ erythrocyte pellets were resuspended in ice-cold 0.2 % NaCl to lyse erythrocytes. After 30 sec, the same volume of 1.6 % NaCl solution was added to reconstitute the isotonic condition. Purified neutrophils were pelleted and then resuspended in a calcium (Ca^{2+})-free Hank's balanced salt solution (HBSS) buffer at pH 7.4, and were maintained at 4 °C before use.

Measurement of Superoxide Anion Generation. The assay of the generation of superoxide anion was based on the SOD-inhibitable reduction of ferricytochrome c. In brief, after supplementation with 0.5 mg/mL ferricytochrome c and 1 mM Ca^{2+}, neutrophils (6×10^5 cells/mL) were equilibrated at 37 °C for 2 min and incubated with drugs or an equal volume of vehicle (0.1 % DMSO, negative control) for 5 min. Cells were activated with 100 nM fMLP during the preincubation of 1 μg/mL cytochalasin B (fMLP/CB) for 3 min. Changes in the absorbance with a reduction in ferricytochrome c at 550 nm were continuously monitored

in a double-beam, six-cell positioner spectrophotometer with constant stirring (Hitachi U-3010, Tokyo, Japan). Calculations were based on differences in the reactions with and without SOD (100 U/mL) divided by the extinction coefficient for the reduction of ferricytochrome c (ε = 21.1/mM/10 mm).

Measurement of Elastase Release. Degranulation of azurophilic granules was determined by elastase release as described previously. Experiments were performed using MeO-Suc-Ala-Ala-Pro-Val-*p*-nitroanilide as the elastase substrate. Briefly, after supplementation with MeO-Suc-Ala-Ala-Pro-Val-*p*-nitroanilide (100 μM), neutrophils (6×10^5/mL) were equilibrated at 37 °C for 2 min and incubated with test compounds or an equal volume of vehicle (0.1 % DMSO, negative control) for 5 min. Cells were activated by 100 nM fMLP and 0.5 μg/mL cytochalasin B, and changes in absorbance at 405 nm were continuously monitored to assay elastase release. The results were expressed as the percent of elastase release in the fMLP/CB-activated, drug-free control system.

Statistical Analysis. All the experiments were performed in triplicate (n=3). Results were expressed as mean ± S.E.M. Calculations of IC_{50} were computer-assisted (PHARM/PCS v.4.2) within the concentrations ranged from 1-10 μg/mL. Statistical comparisons were made between groups using the Student's *t* test. Values of *p* less than 0.05 were considered to be statistically significant, and * $p < 0.05$, ** $p < 0.01$, *** $p < 0.001$, respectively.

Table S1. Inhibitory Effects of extracts from *T. sinensis* on Superoxide Anion Generation and Elastase Release by Human Neutrophils in Response to fMLP/CB

Sample	Superoxide anion generation		Elastase release	
	IC_{50} (μg/mL)[a]	% of inhibition[b]	IC_{50} (μg/mL)[a]	% of inhibition[b]
TS	6.66 ± 0.93	88.07 ± 3.15***	4.68 ± 0.48	73.06 ± 5.50***
TSW	> 10	4.72 ± 0.76**	> 10	0.64 ± 2.41
TSC	5.53 ± 1.95	63.97 ± 6.35**	3.08 ± 0.20	116.32 ± 7.70***
**LY294002** [d]	0.4 ± 0.02***	– [c]	1.5 ± 0.3***	– [c]

[a] Concentration necessary for 50% inhibition (IC_{50}). [b] Percentage of inhibition at 10 μg/mL concentration. Results are presented as mean ± S.E.M. (n=3). ** $p < 0.01$, *** $p < 0.001$ compared with the control value. [c] Not determined. [d] A phosphatidylinositol-3-kinase inhibitor was used as a positive control for superoxide anion generation and elastase release. TS: MeOH extract; TSW: water-soluble layer; TSC: chloroform-soluble layer.

Table S2. Inhibitory Effects of Isolated Compounds on Superoxide Anion Generation and Elastase Release by Human Neutrophils in Response to fMLP/CB

compound	superoxide anion generation	elastase release
	Inh % (10 μM)[a]	Inh % (10 μM)[a]
**1**	20.2 ± 5.1 *	N. A.
**11**	6.7 ± 5.1	6.7 ± 3.8
**14**	3.4 ± 4.2	1.8 ± 4.1
**15**	1.7 ± 0.1***	0.6 ± 2.7
**16**	10.2 ± 7.1	N. A.
**17**	13.8 ± 7.7	N. A.
**24**	6.2 ± 2.7	1.1 ± 3.5
**25**	N. A.	4.7 ± 2.0
**26**	6.5 ± 3.9	N. A.
**27**	3.2 ± 1.0*	0.4 ± 5.6
**39**	6.0 ± 2.4	22.3 ± 10.0
**41**	8.4 ± 3.6	N. A.
**52**	3.5 ± 2.3	2.1 ± 1.7
**53**	4.5 ± 2.3	5.0 ± 1.2*
**54**	1.5 ± 0.2	N. A.
Genistein [b]	84.7 ± 4.2	42.4 ± 3.8

[a] Percentage of inhibition (Inh %) at 10 μM concentration. Results are presented as mean ± S.E.M. (n = 3). * $p < 0.05$, *** $p < 0.001$ compared with the control (fMLP/CB), basal (solvent). [b] Genistein was used as a positive control. N.A.: no inhibition at the tested concentration.

Fig. S1. ^{1}H NMR spectrum of **1**

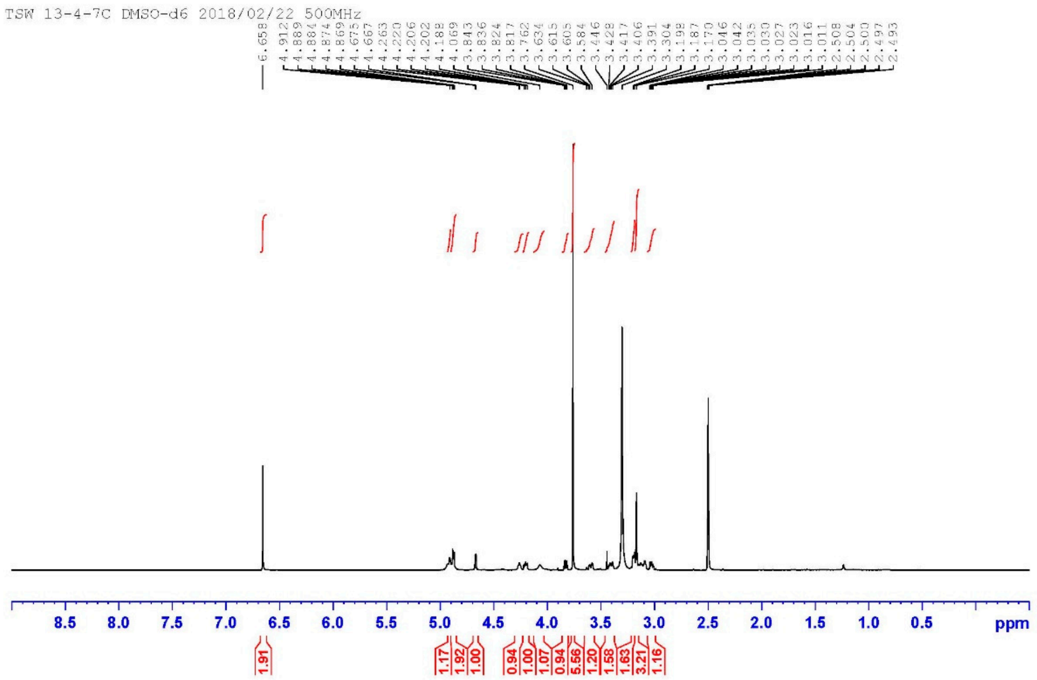

Fig. S2. ^{13}C NMR spectrum of **1**

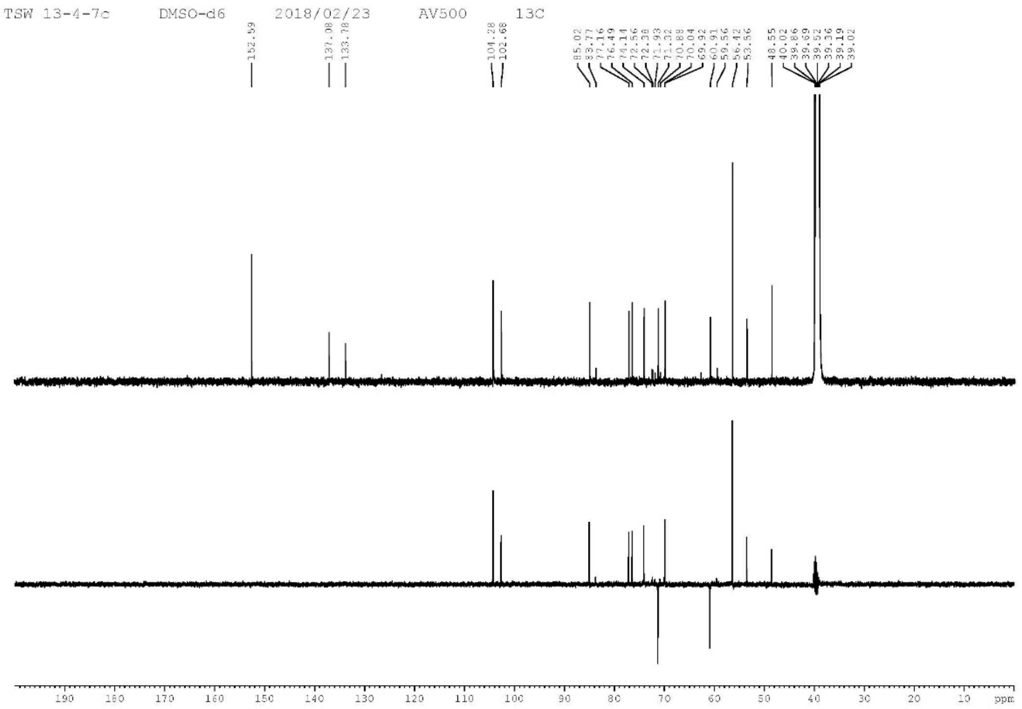

Fig. S3. HMBC spectrum of **1**

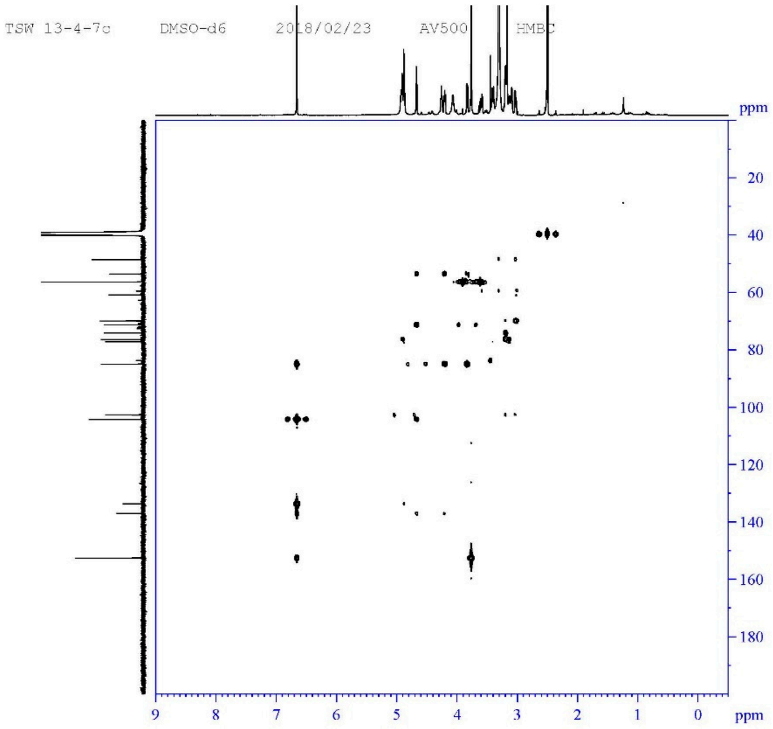

Fig. S4. NOESY spectrum of **1**

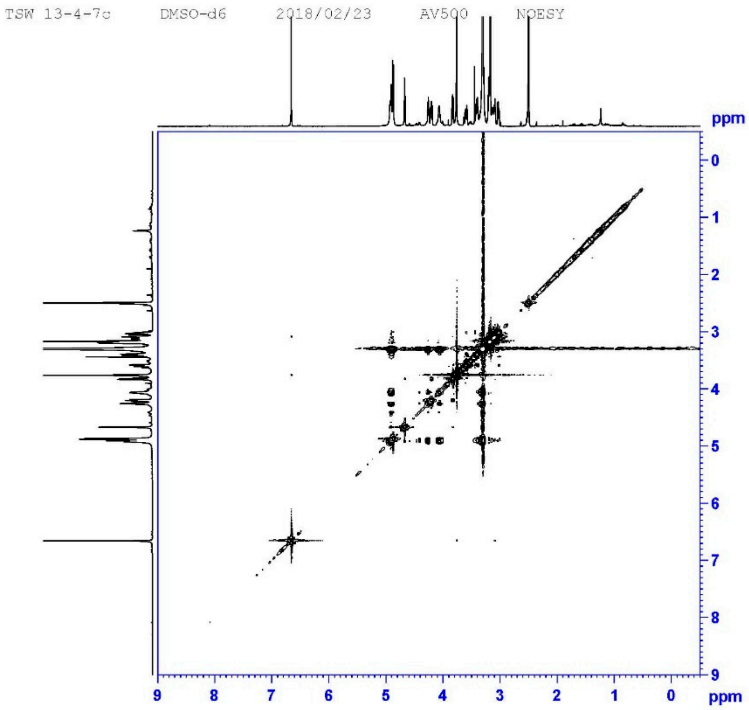

Fig. S5. ^{1}H NMR spectrum of **11**

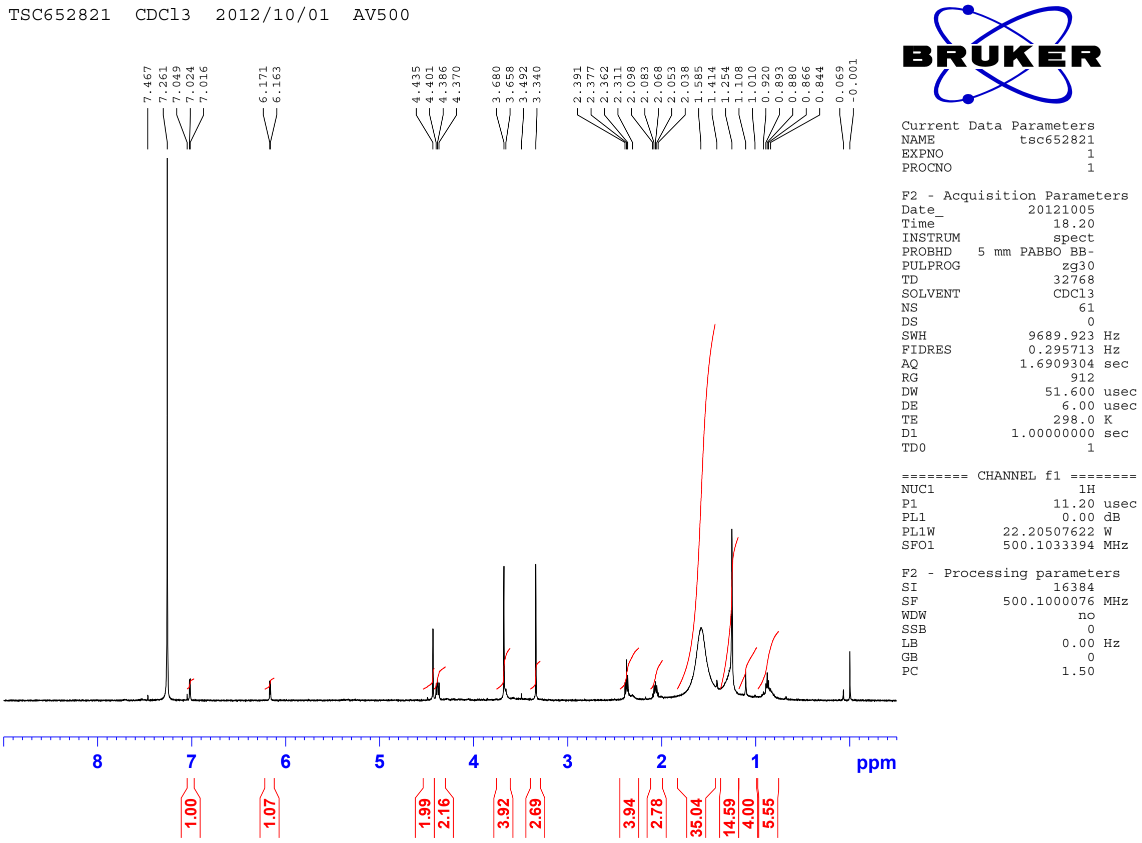

Fig. S6. ^{13}C NMR spectrum of **11**

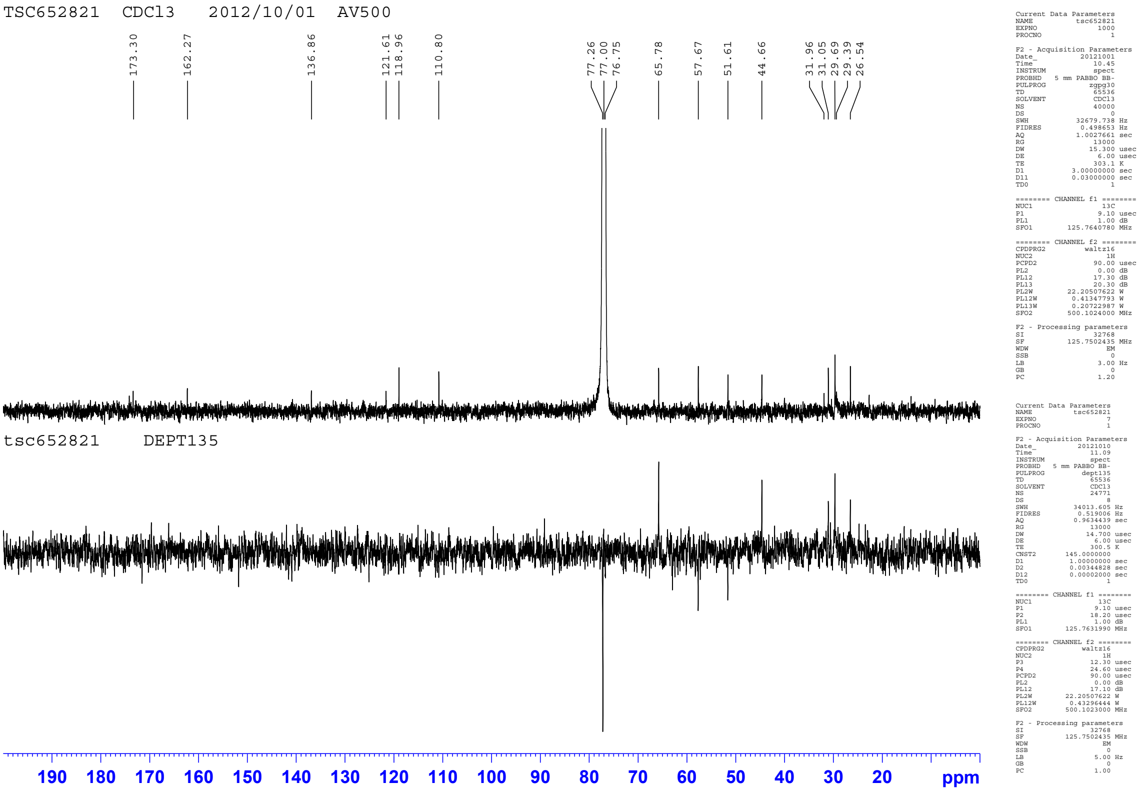

Fig. S7. COSY spectrum of **11**

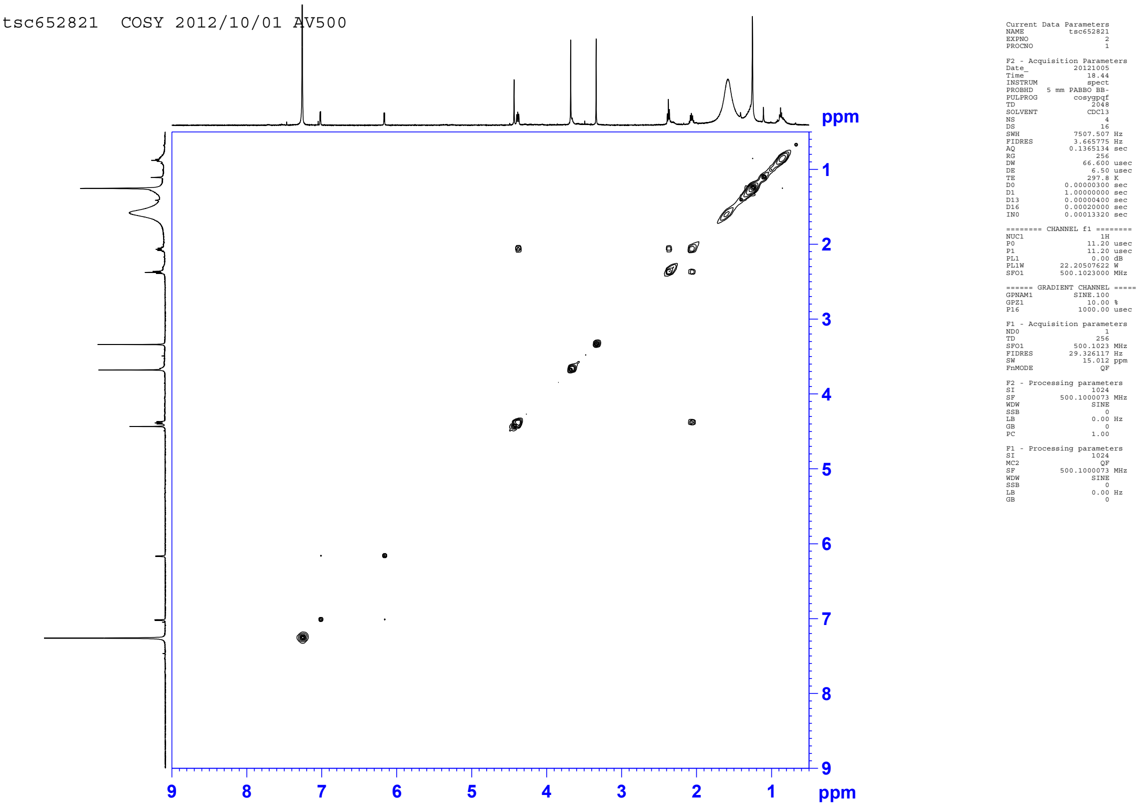

Fig. S8. HMBC spectrum of **11**

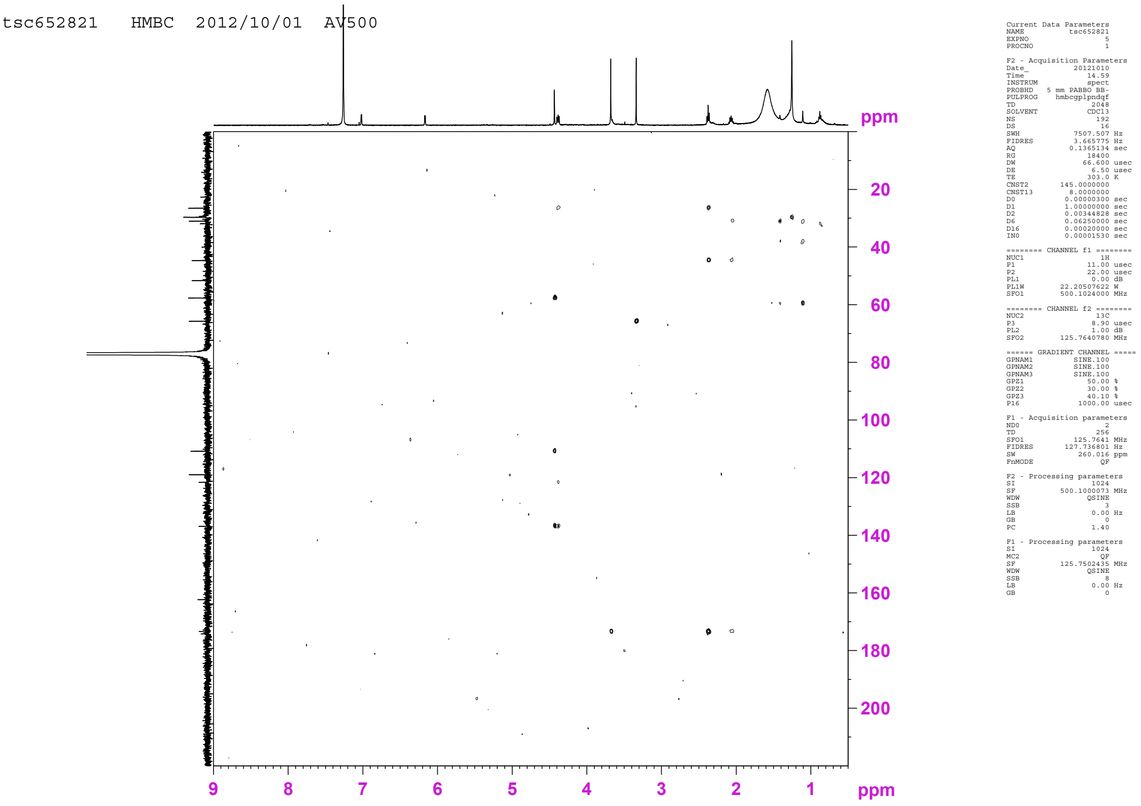

Fig. S9. ^{1}H NMR spectrum of **12**

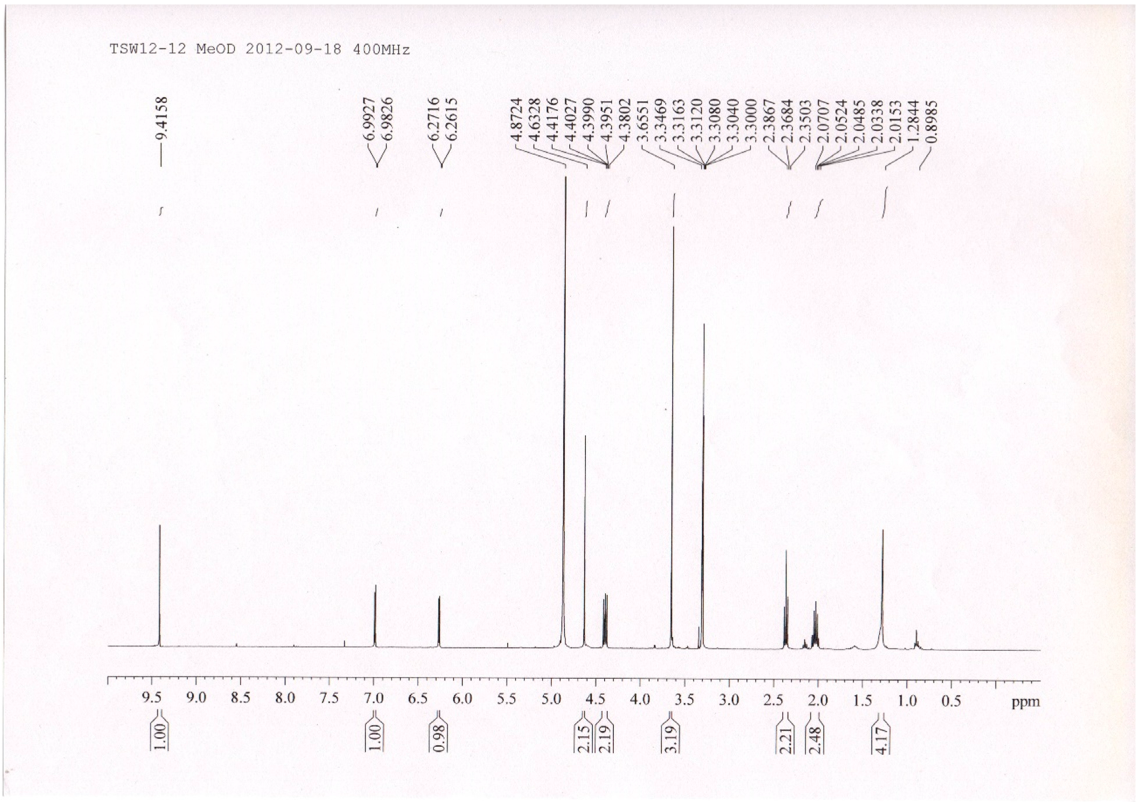

Fig. S10. ^{13}C NMR spectrum of **12**

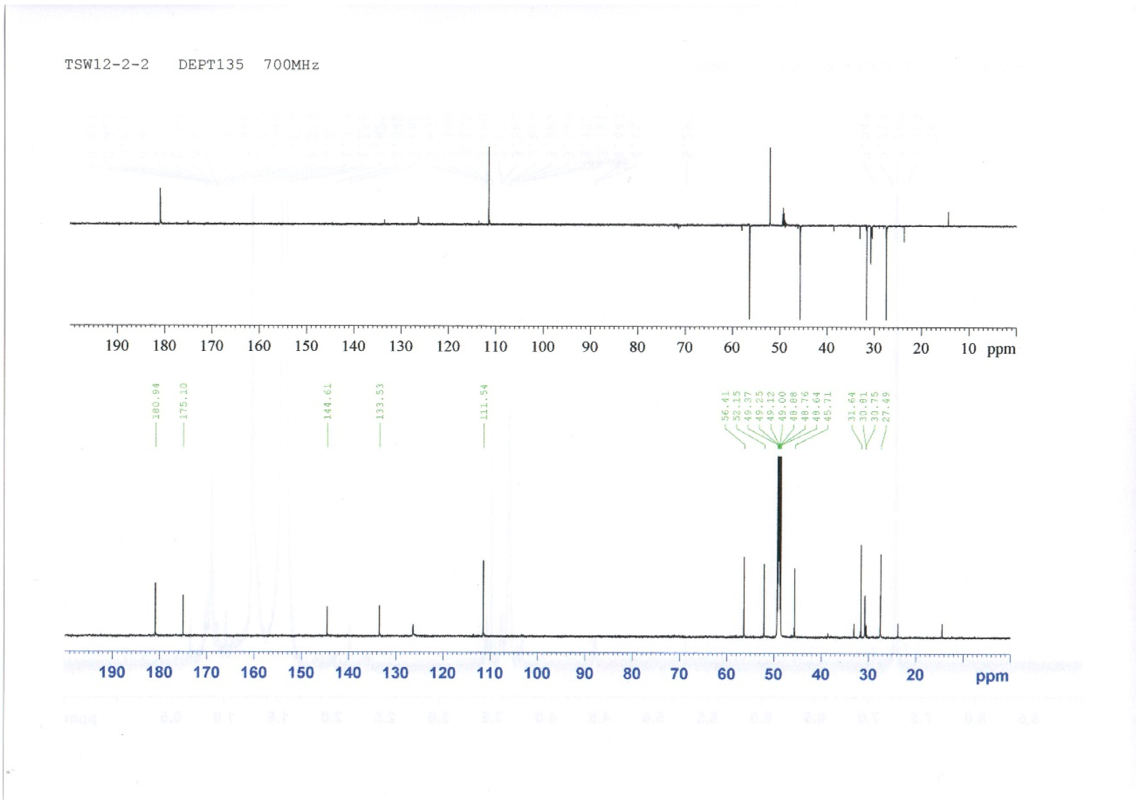

Fig. S11. COSY spectrum of **12**

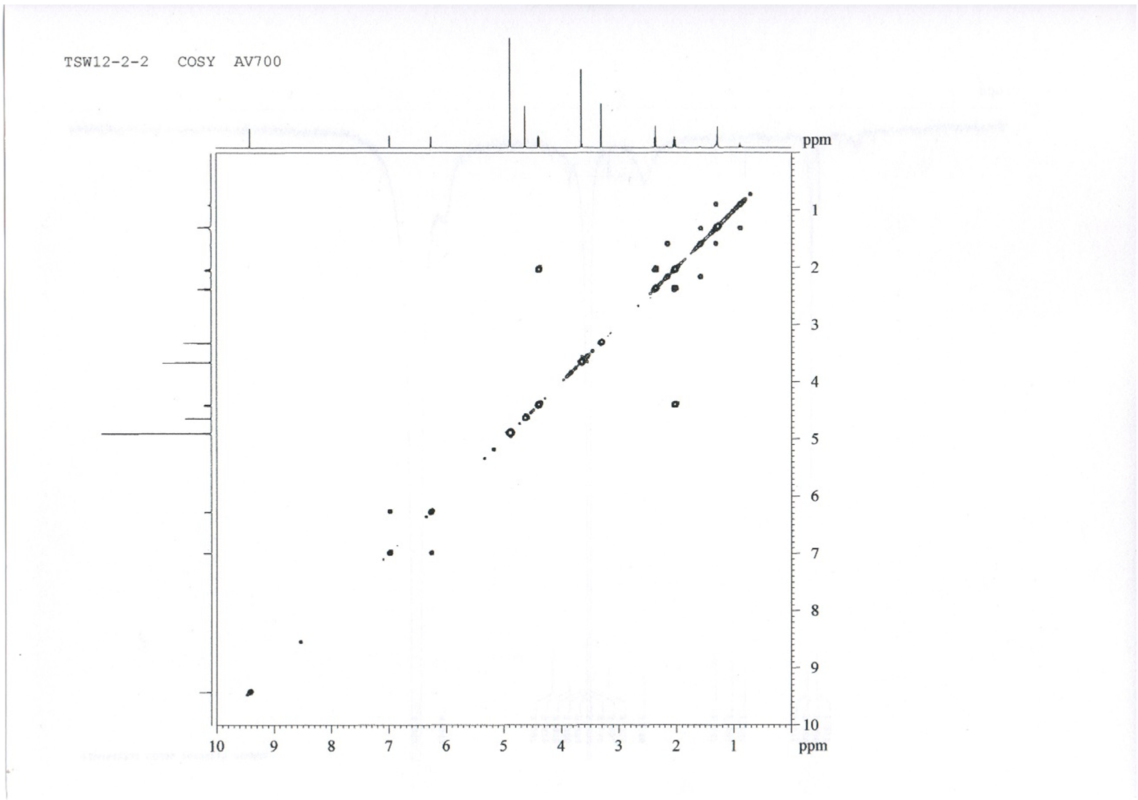

Fig. S12. HMBC spectrum of **12**
